# M&A goodwill and corporate technological innovation: The mediating moderating effect of stock pledges

**DOI:** 10.1371/journal.pone.0271214

**Published:** 2022-08-29

**Authors:** Yan Zhang, Ziyuan Sun, Yiqiang Zhou, Yuting Dong

**Affiliations:** School of Economics and Management, China University of Mining and Technology, Xuzhou, China; Fayoum University Faculty of Computers and Information, EGYPT

## Abstract

Identifying whether and how merger and acquisition (M&A) goodwill influences corporate technology innovation is a critical topic in the context of growing M&A activities. Based on the panel data of 2,634 Chinese non-financial listed companies from 2007 to 2019, this paper adopts multiple linear regression and the Bootstrap method to investigate the impact of M&A goodwill on corporate technological innovation and the mechanism through which M&A goodwill affects technological innovation. The results show that: (1) There is a significant negative correlation between M&A goodwill and technological innovation input, with financial constraints acting as the main channels. (2) Non-private corporations enjoy a higher level of financing capacity in terms of debt and equity compared with private corporations, resulting in the absence of the disincentive effect of M&A goodwill on corporate technological innovation inputs, despite the fact that M&A activities also cause massive resource consumption. (3) Stock pledges by controlling shareholders are effective in alleviating the negative impact of M&A goodwill on technological innovation inputs. Furthermore, our results highlight that simple stock pledge measures capture a significant influence of alleviating financing constraints, thereby decreasing the adverse impact of M&A, and hence need to be adequately considered in future M&A and innovation studies. While enriching the literature on the economic consequences of M&A and the research on stock pledge, this paper also provides micro-evidence for an in-depth understanding of the effect of M&A goodwill on technological innovation.

## 1 Introduction

Innovation is the driving force of economic growth [1]. For most corporations, innovation is an effective way to sustain growth in performance. In order to keep up with the rapid changes in new technologies and products, as well as increasingly fierce technological competition, corporations can no longer rely solely on the “behind closed doors” internal learning and development model to innovate and thus gain a competitive advantage. It is urgent for them to pay great attention to going beyond the boundaries of their organizations and gaining external access to innovative resources. As one of the important approaches to gaining external innovation resources [2], M&A not only alleviates the technology gap, but also provides access to the acquirer’s core technological talent and results in sustained competitiveness [3]. In this context, the goal of M&A is not only to improve financial performance through synergies [4], but to focus more on acquiring high-quality innovative resources to achieve sustainable growth.

**Fig 1:**
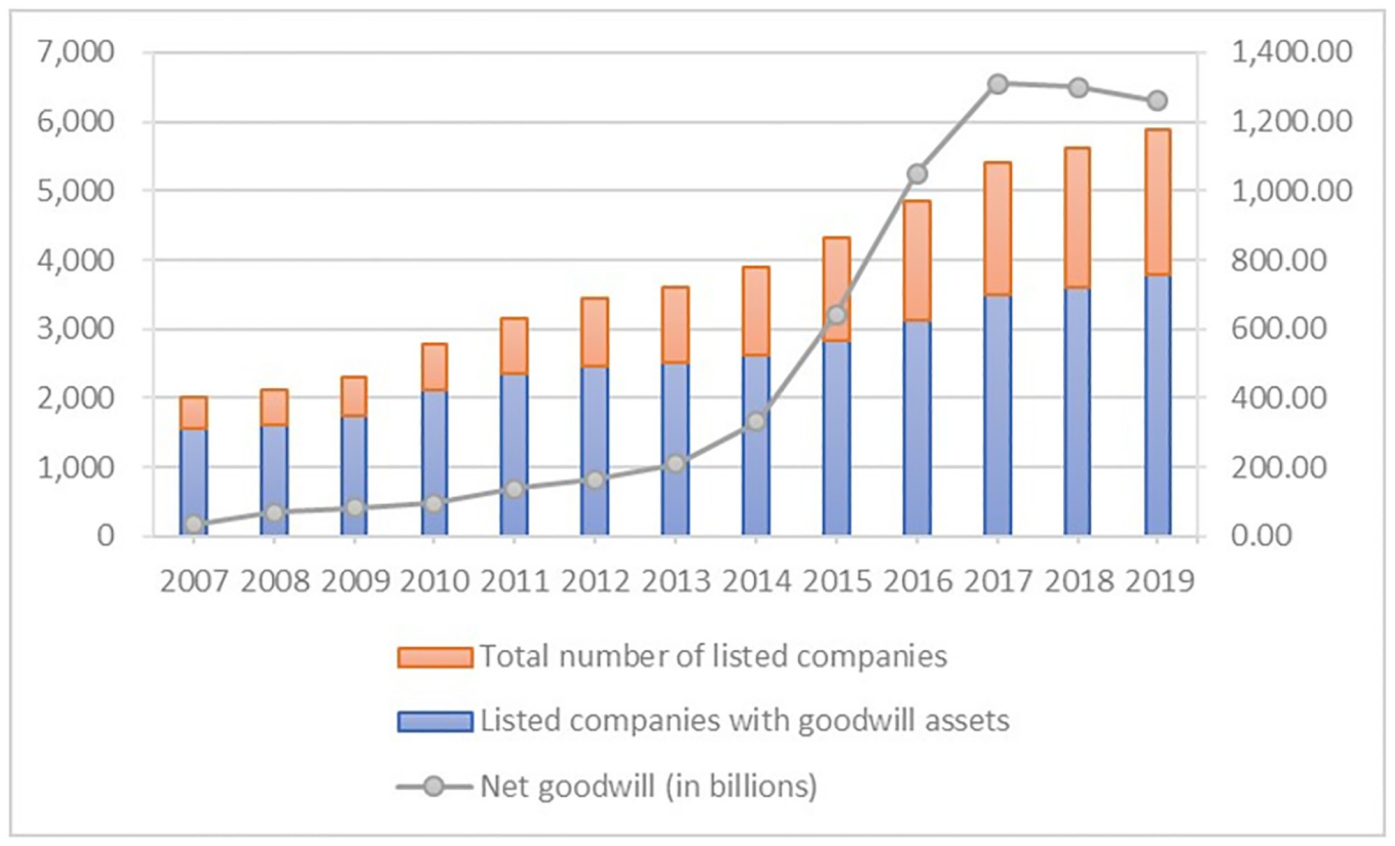
Trend of goodwill of A-share listed corporations in China from 2007 to 2019.

Unfortunately, although M&A can relieve the lack of innovation and inject new energy into the development, it inevitably consumes a great deal of corporate precious resources. As a key accounting term, M&A goodwill reflects the premium paid by the acquirer to the acquiree in a merger or acquisition. The larger the goodwill, the more resources are consumed. In recent years, the scale of goodwill has been rapidly rising, paralleling the increasing frequency of M&A activities. According to the China Stock Market & Accounting Research database, the net value of goodwill of all A-share listed companies in Shanghai and Shenzhen was 330.625 billion yuan in 2014, and then reached 1.26 trillion yuan in 2019, an increase of a total of 1 trillion yuan over 2014, with a growth rate of 380.11%, accounting for a high proportion of 84.16% of the total assets. Fig 1 depicts the trend of goodwill assets of Shanghai and Shenzhen A-shares from 2007 to 2019. If the expected synergy effects are not realized, such a massive scale of goodwill will have a severe influence on corporate production and operations, which in turn will have a negative impact on the enterprise’s innovation.

The related research concerning goodwill mainly focuses on the nature of goodwill, its formation causes, subsequent impairment and economic consequences [5]. Accordingly, these studies are involved in its definition, the relationship between goodwill and firm value, investment efficiency, audit fees, earnings management etc. In various findings of goodwill, the following two diametrically opposed aspects were included: some researchers argue that goodwill reflects the expected synergy effects of M&A and that the two parties involved in M&A can complement each other through M&A and thus improving financial and market performance [5]. In contrast, others state that the formation of M&A goodwill may stem from management’s self-interest motives, including irrational factors in M&A [6][7], thus resulting in a series of negative consequences such as earnings management, goodwill impairment and stock price collapse.

It is apparent from these studies the impact of M&A goodwill on corporate technological innovation and the mechanism are ignored, which are important guides for corporations making M&A decisions. Furthermore, the high amount of goodwill means that corporations will face more severe financing constraints. Controlling shareholders tend to contribute to the formation of large M&A goodwill, and they may subsequently take various measures to ease the financing constraints of the corporation, such as stock pledges. In the context of M&A activities that consume a large amount of corporate resources, stock pledges by controlling shareholders may be quite different from their previous self-interest motives for long-term development. Existing literature generally views stock pledges by controlling shareholders as a tool to hollow out listed companies, neglecting the heterogeneity of stock pledge motives under different scenarios. The impact of stock pledges may vary due to different motives, so the relationship between M&A goodwill, financing constraints and technological innovation will be inconsistent with previous findings.

This paper attempts to address this gap by focusing on the following questions: (1) What are the mechanisms through which M&A goodwill influences corporate technological innovation? (2) Does the difference in financing capacity among corporations with different property rights lead to heterogeneous effects of M&A goodwill on technological innovation? (3) Does controlling shareholders’ stock pledge after the merger and acquisition relieve financing constraints and thus promote technological innovation?

The contributions of this study are in the following three ways: Firstly, this study expands the research horizons of corporate technological innovation. Unlike prior studies that focus on macro and micro-level factors such as macroeconomic policies [8, 9], market competition [10], internal governance [11], and personal traits of executives [12] to explore corporate technological innovation, we incorporate micro-corporate M&A goodwill into this area. Secondly, scholars have carried out extensive and thorough research on the economic consequences of M&A goodwill, but the mechanisms of M&A goodwill are relatively rare. We examine the primary causes of the diverse influence of M&A goodwill on corporate technological innovation under different state ownerships from the perspective of financial constraints. Thirdly, this study provides evidence that stock pledges by controlling shareholders are not always negative. A large number of studies were used to label stock pledges as “bad behavior”. By considering the moderating effect of controlling shareholders’ stock pledges on M&A goodwill and corporate technological innovation in the context of easing corporate financial constraints, we reveal a positive effect of stock pledge at a particular time.

The remainder of this study is organized as follows. Section 2 reviews the prior literature and develops the hypotheses. Section 3 outlines the data and methodology. Section 4 describes the empirical results. Section 5 extends the discussion of our findings. Section 6 offers our concluding remarks.

## 2 Literature review and hypotheses development

### 2.1 M&A goodwill and corporate technological innovation

According to the M&A synergy theory, M&A goodwill reflects the excess earnings capacity of the acquired assets, and the larger the M&A goodwill, the greater the excess profits gained [6, 7]. Healy et al. (1992) [13] and Andrade et al. (2001) [14] discovered that M&A corporations outperform other corporations in the same industry that did not undergo M&A. In terms of market performance, Eckbo (2007) [15] confirmed that the cumulative abnormal returns after the merger and acquisition are usually optimistic, regardless of the acquirer and the acquiree. Goodwill that can realize synergy effects should be a reasonably valued asset. However, the generation of M&A goodwill in China is fraught with too many irrational factors. According to the self-interest hypothesis, the acquiree tends to exaggerate the value and profitability of its assets and conceal the “bad news” of its operating performance or innovation capability before the merger [16]. Unfortunately, due to information asymmetry, the management of the acquirer is unable to effectively screen the actual asset value and development of the acquiree [17], and this professional misjudgment will lead to an excessive premium in the process of M&A. In addition, according to principal-agent theory, managements often have strong incentive to make benefits for themselves. It has been confirmed that regardless of the performance of the post-merger corporation, management’s compensation is reinforced due to the increased size of the corporation [18]. From this point, the acquirer’s management is active in promoting the M&A transaction, resulting in an excessive payment. This kind of M&A goodwill will seriously crowd out the funds that are available for operating activities, and once the cash flow for operating activities is impeded, the corporation will lack enough funds to invest in technological innovation.

In particular, capital markets always react quickly once the expected synergies fail to be achieved, such as a decline in operating performance or a large goodwill impairment [19]. Based on signaling theory, investors may view the corporation more cautiously and be less willing to purchase or hold their shares [20], which in turn will damage cash flow and increase the financial risk. With both high operational and financial risks, the willingness and ability of the acquirer to engage in subsequent risky innovation activities will be significantly decreased, thus inhibiting corporate technological innovation inputs. Based on the above analysis, we propose the following hypothesis:

**H1:** M&A goodwill is negatively correlated with corporate technological innovation inputs.

### 2.2 M&A goodwill, financial constraints and corporate technological innovation inputs

Since Schumpeter proposed the economics of innovation, it has reached a consensus that it is difficult to finance innovative activities in a free competitive market. The output uncertainty, information asymmetry and moral hazard of innovation activities trigger higher external financing costs than internal financing costs [21]. Resource-based theory suggests that financial constraints discouraged corporations from investing in innovation [22]. Considering the heterogeneity of investments, Hottenrott and Peters (2012) argued that financial constraints bind frontier R&D more than conventional R&D [23]. Lee et al. (2015) confirmed that innovative corporations had difficulty accessing financing prior to the financial crisis, and that the problem worsened afterward [24].

The funding for innovation projects mainly stems from two sources: first, external sources of funding, including bank loans and other forms of lending [25]; and second, internal sources of funding, which largely depend on retained earnings and equity. From the perspective of internal funding sources, the high premium paid by the acquirer in the M&A process directly leads to the depletion of the corporate internal funds, and then the funds become unavailable for additional R&D [26]. In terms of external funding, it is also a great challenge for corporations to obtain funds from outside. High M&A goodwill implies higher asset impairment and is more likely to become a “performance bomb” in the future, and the higher the probability of default of the corporation [27]. Consequently, according to signaling theory, high M&A goodwill is equivalent to signaling the market about poor future performance, making investors more rigorous in injecting resources into the corporation. On the one hand, investors may demand a higher return on their investment; on the other hand, rather than investing in poor-quality corporations, they may prefer to lend money to other high-quality corporations in the market [28]. These investor and creditor measures will definitely “add to the woes” of cash-strapped corporations with substantial M&A goodwill.

Additionally, the complexity and uniqueness of innovation projects prevent corporations from disclosing additional information about their innovation efforts on time [29], particularly in the early stages, when observable physical assets are difficult to derive. As a consequence, external investors, such as banks or other creditors, are unaware of the actual progress of R&D activities, and their willingness to lend funds is slim. Both internal and external sources of funds are blocked, which severely aggravates corporate financial constraints, resulting in insufficient funds for innovation activities and thus reducing the corporate technological innovation inputs. Therefore, we propose the second hypothesis of this study:

**H2:** Financial constraints act as an effective mediator between M&A goodwill and corporate technological innovation inputs, specifically, M&A goodwill reduces technological innovation inputs through increasing the degree of financial constraints.

### 2.3 M&A goodwill, state ownership and corporate technological innovation inputs

There is an interesting phenomenon in China’s capital market. Despite the relatively low operational efficiency and capital allocation efficiency of non-private firms, bank credit funds are still over-invested in non-private corporations, i.e., there is “financial discrimination” in ownership [30]. Not only are non-private corporations more likely to obtain loans from banks to ease financial constraints, but their cost of loans is lower. Future more, the government is a powerful supporter of non-private corporations, which means easier access to government assistance, such as more government subsidies [31]. Thus, non-private corporations do not suffer from a lack of innovation capital because of high M&A goodwill.

For private corporations, the existence of ownership “financial discrimination” [30] makes it more difficult for them to obtain loans from banks even under the same condition [32]. In this context, private corporations put greater reliance on access to finance through channels such as commercial credit or private loan [33]. However, these financing methods are generally associated with high risks and high financing costs [34], which do little to alleviate the constraints of corporate financing. With regard to government subsidies, private corporations can also receive fewer government subsidies. Whether it is bank loans or government subsidies, private enterprises do not have the natural advantages of non-private corporations, which limits the development of private corporate innovation, and some technically feasible innovation projects are more likely to be aborted due to lack of funds.

Based on the above analysis, we propose the following hypothesis:

**H3a:** The effect of M&A goodwill on corporate innovation inputs is limited to private corporations and does not extend to non-private corporations.

**H3b:** The difference in financial constraints between private and non-private corporations is the main explanation for the heterogeneous effects of M&A goodwill on corporate technological innovation inputs.

### 2.4 The mediating moderating effect of stock pledges by controlling shareholders

Technological innovation frequently demands a large amount of capital expenditure, and M&A goodwill also consumes a large amount of capital, making corporations anxious for financing. With the implementation of China’s deleveraging policy, bank loans require higher corporate qualifications and longer approval times. To prevent financial risks, banks are adamant about requiring collateral when granting loans [35]. Unlike fixed-asset investments, however, innovative projects lack the ability to provide collateral or security, making it difficult for enterprises to satisfy banks. Typically, private financing is more costly and risky than public financing. In contrast, equity financing is convenient, fast and transferable [36], which can reduce the financing costs and effectively make up for the shortage of private financing. Undoubtedly, there are risks, such as investor misunderstanding, which could result in a stock price crash [37] or a transfer of control [38], thus major shareholders will take comprehensive consideration before stock pledge. When in a relatively stable financial position and the corporation possesses several financing options, controlling shareholders will not choose an aggressive financing method such as stock pledging. Accordingly, when alternative financing channels are blocked and capital needs are urgent, controlling shareholders generally adopt stock pledges to raise funds [38] to help the corporation overcome its difficulties.

Compared with other shareholders, controlling shareholders have the advantage of greater access to information [39] and the ability to effectively monitor and intervene in corporate decisions. Once M&A goodwill depletes corporate resources, controlling shareholders can act as a “supporting hand” for potentially valuable investments in technological innovation [40]. To alleviate the financial pressure on technological innovation investments, stock pledges are regarded as a financing method to raise funds for the corporation. Consequently, when corporate resources are depleted by M&A goodwill and there are no other available financing options, stock pledges by controlling shareholders would effectively reduce the detrimental impact on technological innovation investments. In summary, we propose the fourth hypothesis of this study:

**H4:** Stock pledges by controlling shareholders can alleviate financing constraints and thus weaken the disincentive effect of M&A goodwill on technological innovation input.

**Fig 2:**
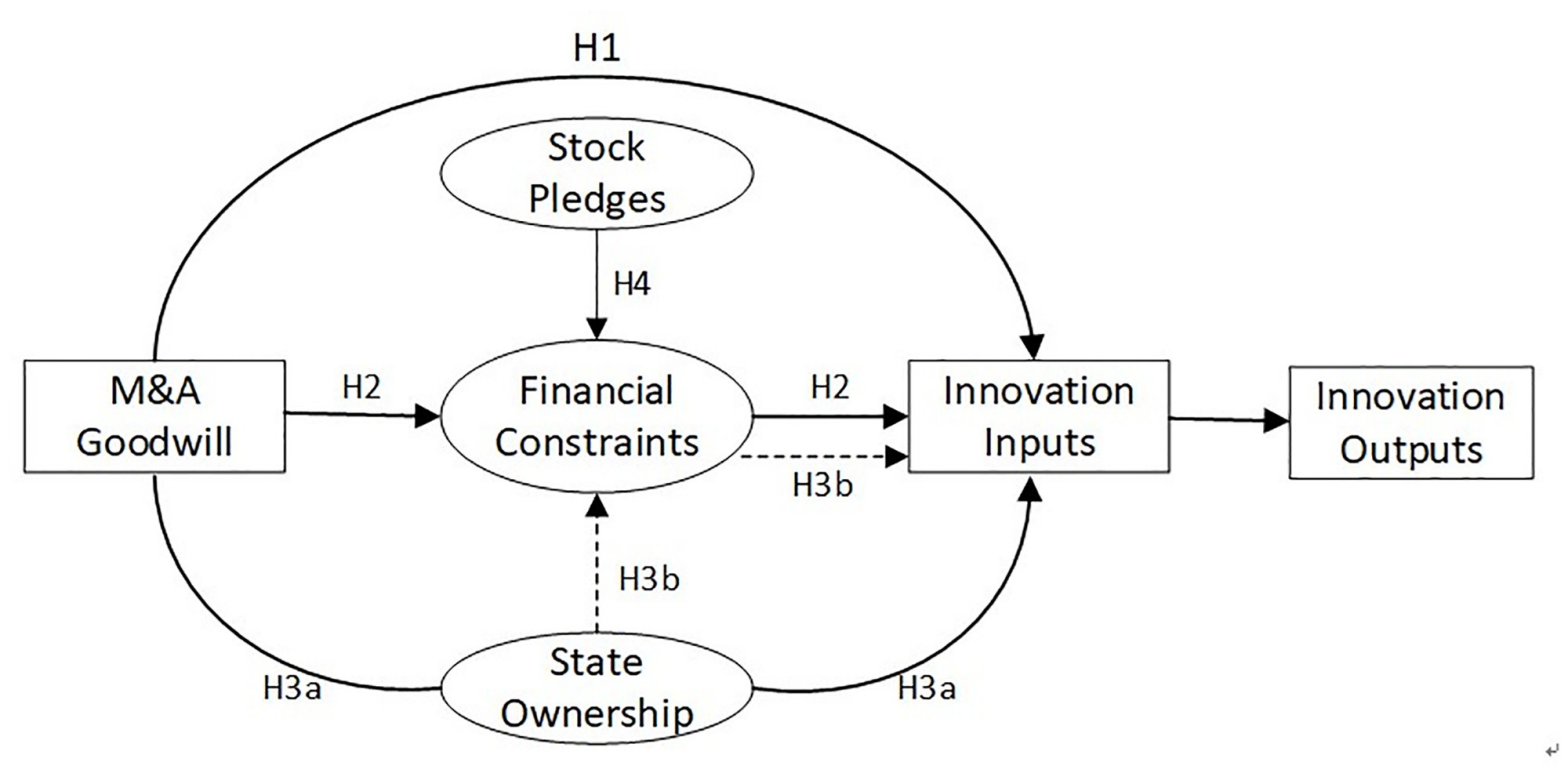
Research framework.

Fig 2 describes the research framework of this paper.

## 3. Sample, variables, and the model

### 3.1 Sample

We focus on all non-financial companies (A-shares) listed on the Shanghai (SSE) and Shenzhen (SZSE) stock exchanges from 2007 to 2019. We select 2007 as the starting point because China started to implement the new accounting standards in 2007. We exclude observations with financial leverage (total liabilities/total assets Lev) of less than 0 or greater than 1, and observations with missing variable data. We retain non-special treatment (ST) firms. All the data for this study comes from the China Stock Market & Accounting Research (CSMAR) database (https://www.gtarsc.com/). The final sample contains 14,186 firm-year observations for 2,634 firms after the initial screening. To control for the effect of outliers, all continuous variables are winsorized at the upper and lower one percentile.

### 3.2 Variables

#### (1) Measures of dependent variable

The explanatory variable in this study is corporate technological innovation inputs (*RD*). We compute it as the ratio of spending on R&D to total assets [41]. Here we need to point out that the existing literature treats missing corporate technological innovation input data in two strategies: some researchers treat the sample of corporations that do not disclose their corporate technological innovation inputs as missing values [42]; while others assume R&D spending for the missing data to be zero [43]. In this study, we treat the sample with missing corporate technological innovation inputs as missing values. For the purpose of robustness testing, we set the missing values of corporate technological innovation inputs to 0.

#### (2) Measures of independent variable

In this study, the explanatory variable is goodwill on acquisitions (*GW*). To measure the difference in M&A goodwill among corporations, this study uses the method of net goodwill divided by total assets at the end of the year. And net goodwill is the difference between consolidated goodwill and the goodwill impairment allowance.

#### (3) Measures of mediating variable

The mediating variable in this study is financial constraints (*FC*). The current measures of financial constraints include the *KZ* index, *WW* index and *SA* index, the first two of which contain asset-liability ratio and cash flow indicators, which are endogenous in nature. The *SA* index can overcome this shortcoming well, as it measures the financial constraints of firms based on two exogenous indicators, namely firm size and firm age [44], and existing studies also show that the SA index can well portray the level of financial constraints of firms. Its specific calculation formula is *SA* = 0.043*Size^2^−0.737*Size−0.04*Age. In this study, the absolute value of the *SA* index is used to indicate the financial constraints (*FC*), and the larger the value, the more severe the financial constraints the firm faces.

#### (4) Measures of moderating variable

The moderating variable in this study is stock pledges by controlling shareholders (*Pledge*). *Pledge* equals 1 if the controlling shareholders had stock pledged by controlling shareholders at the end of the year, and 0 otherwise.

#### (5) Control variables

Following the literature [25, 38], we control factors that could potentially affect corporate innovation, such as leverage ratio (*LEV*), firm size (*Size*), profitability (*Roa*), firm age (*Age*), board independence (*Board*), book-to-market ratio (*Bm*), shareholding of the largest shareholder (*Top*1), sales growth (*Grow*) and state ownership (*Private*). In addition, we treat year and industry as dummy variables in the regressions to control for year and industry fixed effects, respectively. Besides, we present the expected sign between each variable and *RD* based on the related literature, where “+” represents a positive correlation, “-” represents a negative correlation, and “+/-” represents an uncertain sign. See Table 1 for variable definitions.

**Table 1:** Variable definitions.

Names	Expected sign	Descriptions
RD		Ratio of corporate technological innovation inputs to total assets.
GW	-	Net goodwill impairments/ total assets at year end.
FC	-	The absolute value of *SA*.
Pledge	+	Equals 1 if the controlling shareholders had stock pledges at year end, and 0 otherwise.
Size	+	Natural logarithm of total assets at year end.
Lev	-	Ratio of total debt to total assets at year end.
Roa	+	Return on assets, calculated as net income divided by total assets.
Age	-	The natural logarithm of the length of time the corporation is listed.
Board	+	Number of independent directors divided by total number of the board.
Bm	-	Ratio of total assets to market value at year end.
Top1	+/-	Number of shares of the first largest shareholder divided by total share capital.
Grow	+/-	The change in sales revenue from year t-1 to year t divided by sales revenue in year t-1.
Private	-	Equals 1 for private corporations and 0 for non-private corporations.

Source: The Authors.

### 3.3 Model

#### 3.3.1 M&A goodwill and corporate technological innovation

To study the relationship between M&A goodwill and corporate technological innovation inputs, we construct the following model to test H1:

$$\begin{array}{c} RD_{i,t}=\alpha_{0}+\alpha_{1}\times GW_{i,t}+\sum Controls_{i,t}+\epsilon_{i,t} \end{array}$$ (1)

Where *RD*_*i*, *t*_is firm i’s technological innovation inputs in year t, *GW*_*i*, *t*_is firm i’s M&A goodwill in year t, ∑*Controls*_*i*, *t*_represents the set of other control variables that affect firms’ innovation inputs, and *ϵ*_*i*, *t*_denotes the random disturbance term. If *α*_1_is significantly negative, it indicates that higher M&A goodwill inhibits the technological innovation inputs of the corporation.

#### 3.3.2 M&A goodwill, corporate technological innovation and financial constraints: a mediating effect

We use models (2) and (3) to test whether financial constraints are effective mediators between goodwill and technological innovation inputs.

$$\begin{array}{c} FC_{i,t}=\beta_{0}+\beta_{1}\times GW_{i,t}+\sum Controls_{i,t}+\epsilon_{i,t}\;\;\; \end{array}$$ (2)

$$\begin{array}{c} RD_{i,t}=\gamma_{0}+\gamma_{1}\times GW_{i,t}+\gamma_{2}\times FC_{i,t}+\sum Controls_{i,t}+\epsilon_{i,t} \end{array}$$ (3)

Following Baron and Kenny (1986) [45], we use the stepwise regression test coefficient method to verify the existence of mediating effects. In model (2), if the coefficient *β*_1_is significantly positive, it indicates that M&A goodwill exacerbates the corporate financial constraints. In model (3), if the coefficient *γ*_2_is significant, it indicates an indirect effect, and we need to further observe the coefficient of M&A goodwill (*GW*). If the coefficient *γ*_1_is significant, it indicates that the direct effect is significant and there may be other mediators; otherwise, it indicates that the direct effect does not exist and financial constraints (*FC*) are a full mediator.

#### 3.3.3 Stock pledges by controlling shareholders: A mediating moderating effect

In order to test whether stock pledges by controlling shareholders act as a moderator between M&A goodwill and corporate technological innovation inputs, we develop the following stepwise regression model:

$$\begin{array}{cc} FC_{i,t} & =\lambda_{0}+\lambda_{1}\times GW_{i,t}+\lambda_{2}\times Pledge_{i,t}+\lambda_{3}\times GW_{i,t}\times Pledge_{i,t}\;\\ & +\sum Controls_{i,t}+\epsilon_{i,t} \end{array}$$ (4)

$$\begin{array}{cc} RD_{i,t} & =\eta_{0}+\eta_{1}\times GW_{i,t}+\eta_{2}\times Pledge_{i,t}+\eta_{3}\times FC_{i,t}\;\;\;\\ & +\eta_{4}\times FC_{i,t}\times Pledge_{i,t}+\sum Controls_{i,t}+\epsilon_{i,t} \end{array}$$ (5)

According to the hypothesis H4, if the coefficient λ_3_of the interaction term *FC*_*i*, *t*_×*Pledge*_*i*, *t*_in model (4) and the coefficient *η*_3_of the mediating variable *FC*_*i*, *t*_in model (5) are both significantly non-zero, then the first half of the mediating effect is moderated; the second half of the mediating effect is moderated if the coefficient λ_1_of the independent variable *GW*_*i*, *t*_in model (4) and the coefficient *η*_4_of the interaction term *FC*_*i*, *t*_×*Pledge*_*i*, *t*_in model (5) are both significantly non-zero; and the first and second half of the mediating effect is moderated if the coefficient λ_3_of the interaction term *GW*_*i*, *t*_×*Pledge*_*i*, *t*_in model (4) and the coefficient *η*_4_of the interaction term *FC*_*i*, *t*_×*Pledge*_*i*, *t*_in model (5) are both significantly non-zero.

In addition, considering the effect of individual heteroskedasticity on the parameter estimation results, this study uses the estimation method of heteroskedasticity robust standard errors to control for time and industry effects through time and industry dummy variables.

## 4. Empirical results and analysis

### 4.1 Summary statistics

Table 2presents the subgroup summary statistics by state ownership. We can see that in full sample, the mean value of M&A goodwill (*GW*) is 0.0430, and the maximum and minimum values are 0.7518 and 0.0000, respectively. This indicates that the difference of *GW*between different corporations is large. The mean value of corporate technological innovation inputs (*RD*) is 0.0210 greater than the median value (0.0171), which represents corporate technological innovation inputs with strong right bias. And the 75th percentile of *RD*is 0.0280, indicating that the current level of corporate technological innovation inputs of most listed corporations in China is still low. The mean values of financial constraints (*FC*) for private and non-private corporations are 3.7457 and 3.7368 respectively, indicating that private corporations face more severe financial constraints than non-private corporations. For stock pledges, the mean value of stock pledges by controlling shareholders for private corporations is 0.5313, while the mean value for non-private corporations is 0.0883, indicating that private corporations make more controlling shareholders stock pledges. The values and distributions of other variables are basically consistent with existing studies and no significant anomalies are observed in our sample.

**Table 2:** Summary statistics.

Sample	Variables	N	Mean	Sd	P25	P50	P75	Min	Max
**Total**	**GW**	14186	0.0430	0.0897	0.0000	0.0027	0.0371	0.0000	0.7518
	**RD**	14186	0.0210	0.0209	0.0073	0.0171	0.0280	0.0000	0.2723
	**FC**	14186	3.7425	0.2458	3.5869	3.7393	3.8981	2.1177	5.2368
	**Pledge**	14186	0.3710	0.4831	0.0000	0.0000	1.0000	0.0000	1.0000
	**Size**	14186	22.2100	1.2613	21.3323	22.0271	22.8898	18.6744	28.5087
	**Lev**	14186	0.4304	0.2013	0.2724	0.4238	0.5795	0.0075	0.9952
	**Roa**	14186	0.0406	0.0707	0.0149	0.0388	0.0708	-0.9046	1.0895
	**Age**	14186	2.0870	0.7467	1.6094	2.1972	2.7081	0.0000	3.4012
	**Board**	14186	2.1429	0.1955	1.9459	2.1972	2.1972	1.3863	2.8904
	**Bm**	14186	0.6083	0.2464	0.4215	0.6056	0.7902	0.0318	6.5459
	**Top1**	14186	0.3390	0.1444	0.2267	0.3195	0.4318	0.0220	0.8909
	**Grow**	14186	0.2275	0.9173	-0.0087	0.1221	0.2857	-0.9132	33.3695
**Private = 0**	**GW**	5133	0.0143	0.0428	0.0000	0.0002	0.0058	0.0000	0.6121
	**RD**	5133	0.0167	0.0189	0.0026	0.0119	0.0238	0.0000	0.2368
	**FC**	5133	3.7368	0.2963	3.5959	3.7557	3.9134	2.1177	5.2368
	**Pledge**	5133	0.0883	0.2837	0.0000	0.0000	0.0000	0.0000	1.0000
	**Size**	5133	22.7593	1.4777	21.7289	22.5903	23.6504	18.7168	28.5087
	**Lev**	5133	0.5130	0.1990	0.3641	0.5213	0.6653	0.0156	0.9952
	**Roa**	5133	0.0325	0.0586	0.0091	0.0283	0.0567	-0.5204	0.3507
	**Age**	5133	2.4869	0.6161	2.1972	2.7081	2.9444	0.0000	3.4012
	**Board**	5133	2.2184	0.1905	2.1972	2.1972	2.3026	1.6094	2.8904
	**Bm**	5133	0.6681	0.2699	0.4685	0.6721	0.8823	0.0523	6.5459
	**Top1**	5133	0.3849	0.1535	0.2641	0.3778	0.4961	0.0362	0.8909
	**Grow**	5133	0.1737	0.8587	-0.0363	0.0866	0.2236	-0.8625	25.2582
**Private = 1**	**GW**	9053	0.0593	0.1041	0.0000	0.0080	0.0717	0.0000	0.7518
	**RD**	9053	0.0235	0.0216	0.0105	0.0194	0.0298	0.0000	0.2723
	**FC**	9053	3.7457	0.2118	3.5828	3.7239	3.8785	3.3901	4.8015
	**Pledge**	9053	0.5313	0.4990	0.0000	1.0000	1.0000	0.0000	1.0000
	**Size**	9053	21.8985	0.9934	21.1875	21.8012	22.5130	18.6744	26.4966
	**Lev**	9053	0.3836	0.1871	0.2341	0.3760	0.5172	0.0075	0.9947
	**Roa**	9053	0.0452	0.0763	0.0196	0.0457	0.0771	-0.9046	1.0895
	**Age**	9053	1.8603	0.7187	1.3863	1.9459	2.3026	0.0000	3.4012
	**Board**	9053	2.1001	0.1850	1.9459	2.1972	2.1972	1.3863	2.8332
	**Bm**	9053	0.5744	0.2251	0.4027	0.5766	0.7430	0.0318	1.4626
	**Top1**	9053	0.3130	0.1322	0.2130	0.2966	0.3964	0.0220	0.8824
	**Grow**	9053	0.2579	0.9475	0.0067	0.1459	0.3173	-0.9132	33.3695

### 4.2 Regression analysis

#### 4.2.1 Verify hypothesis H1

Model 1 and Model 2 in Table 3present the regression results of hypothesis H1. By comparing them, it can be seen that the sign and significance of the control variable coefficients do not change substantially when the independent variable is introduced, and the adj.*R*^2^improves, indicating that the variable selection in this study is reasonable. The coefficient of *GW*in Model 2 is -0.0243 (t=-6.75), which means M&A goodwill has a substantial, negative relationship with *RD*. This finding supports the resource-based theory, that corporations reduce technological innovation inputs as M&A goodwill increases. Accordingly, hypothesis H1 holds. Concerning control variables, the coefficients of *Lev*, *Age*and *Bm*all have a significant negative relationship with *RD*, which is consistent with prior research[46–48]. Moreover, the coefficient of Roa is significantly positive, corroborating the study of Yuan and Wen (2018) [47], who posited that corporations with greater profitability are more likely to engage in innovative activities.

**Table 3:** Regression results of hypothesis H1, H2 and H3.

Variables	Full Sample				Private = 1	Private = 0
	Model 1	Model 2	Model 3	Model 4	Model 5	Model 6
	*RD*_*t*_	*RD*_*t*_	*FC*_*t*_	*RD*_*t*_	*RD*_*t*_	*RD*_*t*_
**GW**		−0.0243*** (-6.75)	0.1058*** (2.72)	−0.0239*** (-6.71)	−0.0284*** (-7.28)	0.0152 (1.25)
**FC**				−0.0035* (-2.48)		
**Size**	0.0003 (0.82)	0.0004 (1.04)	−0.0796*** (-9.17)	0.0001 (0.30)	0.0014* (1.99)	−0.0009* (-2.18)
**Lev**	−0.0031* (-1.86)	−0.0047*** (-2.85)	0.0903*** (3.88)	−0.0044*** (-2.64)	−0.0063*** (-2.95)	-0.0038 (-1.44)
**Roa**	0.0253*** (5.56)	0.0236*** (5.27)	0.3902*** (7.67)	0.0249*** (5.44)	0.0200*** (3.88)	0.0307*** (3.81)
**Age**	−0.0021*** (-4.22)	−0.0022*** (-4.44)	0.1541*** (23.90)	−0.0016*** (-2.88)	−0.0026*** (-3.38)	−0.0031*** (-3.46)
**Board**	0.0015 (0.77)	0.0012 (0.60)	0.0543*** (2.62)	0.0014 (0.68)	0.0032 (1.14)	-0.0012 (-0.40)
**Bm**	−0.0207*** (-8.83)	−0.0206*** (-8.91)	0.1798*** (8.12)	−0.0200*** (-8.74)	−0.0282*** (-10.42)	−0.0112*** (-4.02)
**Top1**	-0.0015 (-0.67)	-0.0033 (-1.45)	−0.1019*** (-3.03)	-0.0036 (-1.60)	−0.0047* (-1.67)	-0.0006 (-0.17)
**Grow**	−0.0003* (-2.22)	-0.0001 (-0.39)	0.0011 (0.74)	-0.0001 (-0.37)	-0.0000 (-0.21)	-0.0000 (-0.17)
**Constant**	0.0056 (0.72)	0.0058 (0.75)	4.7177*** (29.47)	0.0222* (2.18)	-0.0134 (-1.00)	0.0346*** (3.57)
**Ind**	Yes	Yes	Yes	Yes	Yes	Yes
**Year**	Yes	Yes	Yes	Yes	Yes	Yes
**N**	14186	14186	14186	14186	9053	5133
**adj. *R*^2^**	0.281	0.289	0.359	0.290	0.272	0.304

Notes: T-statistics in parentheses are the basis of standard errors clustered by firms and robust to heteroscedasticity.

“***”, “*”, and “*” respectively denote the significance on the basis of two-tailed t-tests at or below 1%, 5%, and 10% levels.

#### 4.2.2 Verify hypothesis H2

Model 3 and Model 4 in Table 3show the regression results of hypothesis H2. In Model 3, the regression coefficient of M&A goodwill (*GW*) on financial constraints (*FC*) is 0.1058 and significant at the 1% level (p = 0.000), indicating that M&A goodwill significantly increases corporate financial constraints, and the expected synergy effects fail to be achieved [49]. This finding implies that the higher the premium paid for a M&A, the more corporate resources are consumed and, as a result, the greater the financing constraints that the corporation faces. Besides, in Model 4, the coefficient of financial constraints (*FC*) on corporate technological innovation inputs (*RD*) is -0.0035 (p = 0.013), implying that M&A goodwill raises financing constraints and inevitably impedes innovation activity, which is in line with Brown et al. (2012) [50]. In terms of M&A goodwill (*GW*), the coefficient is -0.0239 and significant at the 1% level (p = 0.000), which indicates that financial constraints are a partial mediator between M&A goodwill and corporate technological innovation inputs. Thus, hypothesis H2 is confirmed.

#### 4.2.3 Verify hypothesis H3

H3a proposes that state ownership changes the disincentive effect of M&A goodwill on the performance of corporate technological innovation inputs. Model 5 and Model 6 in Table 3presents the results. For private corporations (Private = 1), the regression coefficient of M&A goodwill (*GW*) is -0.0284 and significant at the 1% level (t=-7.28) in the current period of M&A, which indicates that in private corporations, M&A goodwill significantly inhibits their subsequent technological innovation inputs due to the shortage of resources [49]. While for non-private corporations (Private = 0), the coefficient of GW is positive but insignificant (t = 1.25), indicating that the disincentive effect of M&A goodwill on technological innovation inputs does not exist. Therefore, hypothesis H3a is verified.

Before validating H3b, we ran the mean difference and median difference tests for all variables according to state ownership, and the results are shown in Table 4. The mean of financial constraints (*FC*) for private corporations is 3.746, which is 0.009 higher than the mean of non-private corporations, and the mean difference is significant at the 5% level, indicating that Chinese private corporations face more severe financial constraints, which is similar to the findings of Cull et al. (2015) [51]. Additionally, all variables are significantly different in both the mean and median. This provides a basis for further differentiation and comparison of the diverse impacts of private and non-private corporations.

**Table 4:** Univariate tests.

Variables	Private = 0			Private = 1			Diff	
	N	Mean	Median	N	Mean	Median	MeanDiff	MedianDiff
**GW**	5133	0.014	0.000	9053	0.059	0.008	−0.045***	1010.093***
**RD**	5133	0.017	0.012	9053	0.024	0.019	−0.007***	473.189***
**FC**	5133	3.737	3.756	9053	3.746	3.724	−0.009**	38.040***
**Pledge**	5133	0.088	0.000	9053	0.531	1.000	−0.443***	2755.565***
**Size**	5133	22.759	22.590	9053	21.899	21.801	0.861***	724.939***
**Lev**	5133	0.513	0.521	9053	0.384	0.376	0.129***	868.813***
**Roa**	5133	0.033	0.028	9053	0.045	0.046	−0.013***	396.044***
**Age**	5133	2.487	2.708	9053	1.860	1.946	0.627***	2595.694***
**Board**	5133	2.218	2.197	9053	2.100	2.197	0.118***	1059.304***
**Bm**	5133	0.668	0.672	9053	0.574	0.577	0.094***	195.867***
**Top1**	5133	0.385	0.378	9053	0.313	0.297	0.072***	485.561***
**Grow**	5133	0.174	0.087	9053	0.258	0.146	−0.084***	195.867***

**Fig 3:**
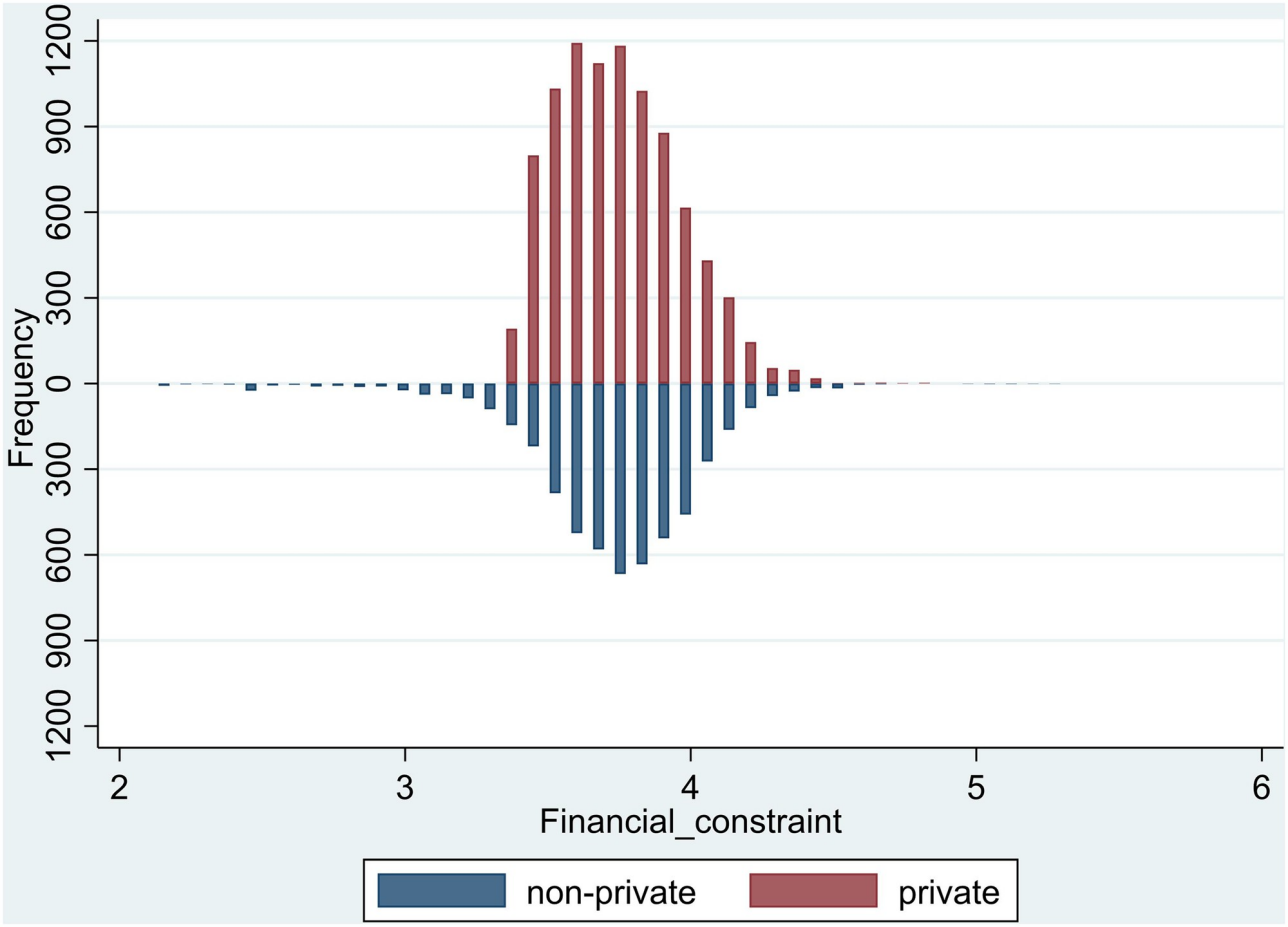
Distribution frequency of financial constraints in private and non-private corporations.

In addition, Fig 3depicts the distribution of financial constraints for private and non-private corporations. As can be seen, when financial constraints are at a low level, some non-private corporations are mainly dispersed, when financial constraints are severe, private corporations are primarily spread. This further indicates that private corporations are subject to greater financial constraints than non-private corporations.

Panel A and Panel B of Table 5present the regression results of the mediating effects for private and non-private corporations, respectively. For private corporations (Private = 1), the regression coefficient of M&A goodwill (*GW*) on financial constraints (*FC*) in Model 2 is 0.0917 and significant at the 5% level (p = 0.021), while the regression coefficients of *FC*and *GW*in Model 3 are both significantly negative, which indicates that financial constraints are a partial mediator of M&A goodwill and technological innovation inputs hold in private corporations. This phenomenon is due to the weak political connections between private corporations and the government [51]. On the contrary, in non-private corporations (Private = 0), while the regression coefficient of *GW*on financial constraints (*FC*) in Model 5 is 0.3525 and significant at the 1% level (p = 0.005), the regression coefficient of financial constraints (*FC*) on technological innovation inputs (*RD*) in Model 6 is significantly negative at the 10% level, with the opposite coefficient sign to M&A goodwill (*GW*) and thus the mediating effect is not available. That is, for non-private corporations, financial constraints cannot be an effective mediator between M&A goodwill and technological innovation inputs. In general, the heterogeneity of financial constraints between private and non-private corporations is the primary explanation for the diverse effects of M&A goodwill on corporate technological innovation inputs, and hypothesis H3b is confirmed.

**Table 5:** Analysis of the mediating effects of financial constraints under different state ownerships.

Variables	Private = 1			Private = 0		
	Model 1	Model 2	Model 3	Model 4	Model 5	Model 6
	*RD*_*t*_	*FC*_*t*_	*RD*_*t*_	*RD*_*t*_	*FC*_*t*_	*RD*_*t*_
**GW**	−0.0284*** (-7.28)	0.0917** (-7.28)	−0.0280*** (-7.28)	0.0152 (-7.28)	0.3525*** (-7.28)	0.0162 (-7.28)
**FC**			−0.0042** (-2.04)			−0.0031* (-1.70)
**Size**	0.0014** (1.99)	−0.0163*** (-2.72)	0.0013* (1.89)	−0.0009** (-2.18)	−0.1129*** (-10.12)	−0.0013*** (-2.61)
**Lev**	−0.0063*** (-2.95)	0.0203 (0.86)	−0.0062*** (-2.91)	-0.0038 (-1.44)	0.1476*** (3.61)	-0.0033 (-1.25)
**Roa**	0.0200*** (3.88)	0.1599*** (3.80)	0.0207*** (3.97)	0.0307*** (3.81)	0.6759*** (3.81)	0.0328*** (3.95)
**Age**	−0.0026*** (-3.38)	0.1120*** (17.35)	−0.0021** (-2.54)	−0.0031*** (-3.46)	0.2110*** (14.64)	−0.0025*** (-2.71)
**Board**	0.0032 (1.14)	0.0482** (2.28)	0.0034 (1.21)	-0.0012 (-0.40)	0.0926** (2.51)	-0.0009 (-0.30)
**Bm**	−0.0282*** (-10.42)	0.1204*** (5.37)	−0.0277*** (-10.39)	−0.0112*** (-4.02)	0.1971*** (5.21)	−0.0106*** (-3.89)
**Top1**	−0.0047* (-1.67)	0.0373 (1.13)	-0.0045 (-1.61)	-0.0006 (-0.17)	−0.2075*** (-3.28)	-0.0012 (-0.35)
**Grow**	-0.0000 (-0.21)	0.0011 (0.53)	-0.0000 (-0.18)	-0.0000 (-0.17)	0.0007 (0.28)	-0.0000 (-0.16)
**Constant**	-0.0134 (-1.00)	3.5354*** (27.22)	0.0016 (0.11)	0.0346*** (3.57)	5.1815*** (24.83)	0.0505*** (3.66)
**Ind**	Yes	Yes	Yes	Yes	Yes	Yes
**Year**	Yes	Yes	Yes	Yes	Yes	Yes
**N**	9053	9053	9053	5133	5133	5133
**adj. *R*^2^**	0.272	0.296	0.274	0.304	0.532	0.305

#### 4.2.4 Verify hypothesis H4

Table 6reports the results of hypothesis H4. The coefficient of the interaction term *GW*_*Pledge*between M&A goodwill and stock pledges by controlling shareholders is negative at -0.0995 and significant at the 10% level in Model 1. This means that whereas goodwill raises corporate financing constraints [26], it can be effectively mitigated by controlling shareholders’ stock pledges in the context of premium M&A. Meanwhile, the coefficient of the mediating variable *FC*in Model 2 is negative at -0.0045 and significant at the 1% level. Overall, the coefficients of the interaction term *GW*_*Pledge*and the mediating variable *FC*are both significant and non-zero, indicating that the stock pledges by controlling shareholders effectively mitigates the first half of the path in which M&A goodwill affects corporate technological innovation inputs by exacerbating corporate financial constraints, and the moderating mediating effect exists. “Supporting hand” theory for the controlling shareholder does actually appear under special circumstances [40, 52]. Hypothesis H4 is confirmed.

**Table 6:** Moderated mediation test for stock pledges by controlling shareholders.

Variables	Model 1	Model 2
	*FC*_*t*_	*RD*_*t*_
**GW**	0.1492*** (2.71)	−0.0085*** (-2.79)
**Pledge**	0.0025 (0.37)	-0.0044 (-0.56)
**GW_Pledge**	−0.0995* (-1.72)	
**Soe**	0.0212** (2.03)	0.0005 (0.52)
**FC**		−0.0045*** (-2.81)
**FC_Pledge**		0.0007 (0.35)
**Constant**	4.7121*** (29.07)	0.0535*** (4.53)
**Controls**	Yes	Yes
**Year**	Yes	Yes
**N**	14186	14186
**adj. *R*^2^**	0.359	0.291

### 4.3 Endogeneity

Corporations with poor innovation may engage in M&A activity to improve their innovation level, so the regression result can be interpreted as a reverse causality between M&A goodwill and technological innovation. Fortunately, the instrumental variable method can solve this problem perfectly. Taking the goodwill of M&A with one lag period as the instrumental variable (*IV*), and then making a 2*SLS*regression. The validity test in the first stage shows that Shea’s Partial *R*_*sq*value is 0.7620, and the F statistic is 10079.80 (p = 0.000), which is much more than 10, indicating that the selected instrumental variable is valid. The regression results are shown in Table 7. It can be found that after considering endogenous problems, M&A goodwill remains significantly restrains corporate technological innovation inputs, which is consistent with the previous findings.

**Table 7:** Regression analysis results of endogeneity with 2SLS.

Variables	Model 1	Model 2	Model 3
	*GW*_*t*_	*RD*_*t*_	*RD*_*t*+ 1_
**IV**	0.8313*** (100.38)		
**GW**		−0.0152*** (-5.26)	−0.0116*** (-3.06)
**Constant**	0.0452*** (3.99)	0.0237*** (4.87)	0.0296*** (5.16)
**Controls**	Yes	Yes	Yes
**Ind**	Yes	Yes	Yes
**Year**	Yes	Yes	Yes
**N**	10689	10689	7916
**adj. *R*^2^**	0.762	0.298	0.288
**Cragg-Donald Wald F**	10079.80	—	—

### 4.4 Robustness tests

The following five methods are used for robustness tests.

Firstly, other indicators to measure the dependent variable. The natural logarithm of the amount of corporate technological innovation inputs was used to re-measure technological innovation inputs, with the regression results held constant. The regression results are shown in S1 Appendix.

Secondly, observe the longer-term effects. The dependent variable RD is re-regressed with one and two lags, respectively, and the regression results are shown in S2 Appendix. It can be seen that the coefficient between M&A goodwill and corporate innovation remains significantly negative at one and two lags in the full sample. The regression coefficient of M&A goodwill remains significantly negative after two lags for private corporations (Private = 1), whereas it is not significant for non-private corporations (Private = 0) with one or two lags. This further suggests that the inhibitory effect of M&A goodwill on corporate technological innovation is only appropriate for private corporations.

Thirdly, the robustness of the mediating effect is tested by replacing other indicators to measure financial constraints. We use the KZ index to re-measure the financial constraints. Then, stepwise regressions were used for the full sample of private and non-private corporations, respectively, and the regression results are shown in S3 Appendix. It can be seen that the findings of the study remain consistent with the previous sections.

Fourthly, this study assigns the missing values of corporate technological innovation inputs to 0 for robustness testing, and the regression results are shown in S4 Appendix.

Fifthly, in order to ensure the robustness of hypothesis H4, this study uses the Bootstrap method based on deviation correction to directly calculate the confidence interval of the product of coefficients, tests the robustness of the mediating effect with adjustment, and repeats 5000 times. The results are shown in Table S5 Appendix. It can be seen that the indirect effect of M&A goodwill on technological innovation inputs through financial constraints is regulated by the stock pledge of controlling shareholders, and the coefficient product term does not include 0 in the 95% confidence interval. Therefore, the interval test of the product of coefficients by using the Bootstrap method is consistent with the results of the above mixed sequential regression test.

## 5. Further discussion: The impact on corporate technological innovation outputs

To further explore the impact of M&A goodwill on the technological innovation outputs, we measure the technological innovation outputs from two aspects, namely, patent application and patent authorization. The indicators of patent application fall into three categories: the total number of patent application (*pat*_*apply*_*all*), the total number of invention patent application (*pat*_*apply*_*in*) and the total number of non-invention patent application (*pat*_*apply*_*noin*) (including utility model and design). Accordingly, the indicators of patent authorization fall into three categories: the total number of patents granted (*pat*_*obtain*_*all*), the total number of invention patents granted (*pat*_*obtain*_*in*) and the total number of non-invention patents granted (*patent*_*obtain*_*noin*). The regression results are shown Table 8.

**Table 8:** Regression results of M&A goodwill and corporate technological innovation outputs.

Variables	Model 1	Model 2	Model 3	Model 4	Model 5	Model 6
	*pat*_*apply*_*all*	*pat*_*apply*_*in*	*pat*_*apply*_*noin*	*pat*_*obtain*_*all*	*pat*_*obtain*_*in*	*patent*_*obtain*_*noin*
**GW**	−1.0969*** (-4.46)	−0.9300*** (-3.98)	−0.7806*** (-3.98)	−1.0955*** (-4.75)	−0.9576*** (-4.96)	−0.8408*** (-3.76)
**Size**	0.6289*** (19.41)	0.6437*** (19.90)	0.5309*** (16.44)	0.5802*** (17.98)	0.5322*** (17.38)	0.5224*** (15.96)
**Lev**	−0.2646* (-1.88)	−0.2953** (-2.23)	-0.0703 (-0.51)	-0.2157 (-1.60)	−0.4121*** (-3.68)	-0.0833 (-0.61)
**Roa**	1.0637*** (3.58)	0.6659** (2.45)	0.8387*** (2.87)	0.6781** (2.36)	-0.0124 (-0.05)	0.7159** (2.41)
**Age**	−0.1542*** (-3.56)	−0.1420*** (-3.56)	−0.1303*** (-3.08)	−0.1058** (-2.54)	-0.0407 (-1.22)	−0.0980** (-2.32)
**Board**	0.0032 (0.02)	0.1258 (1.01)	-0.1012 (-0.79)	-0.0261 (-0.20)	0.1088 (1.02)	-0.1171 (-0.90)
**Bm**	−0.5357*** (-4.35)	−0.8988*** (-6.82)	−0.2406** (-2.07)	−0.4654*** (-3.94)	−0.7723*** (-6.53)	−0.2294** (-1.97)
**Top1**	−0.4463** (-2.28)	−0.4391** (-2.42)	-0.2480 (-1.32)	−0.4197** (-2.23)	-0.1939 (-1.25)	−0.3495* (-1.86)
**Grow**	-0.0131 (-1.11)	-0.0121 (-1.01)	-0.0133 (-1.20)	−0.0287** (-2.34)	-0.0147 (-1.57)	−0.0302** (-2.37)
**Constant**	−12.0606*** (-18.11)	−13.0403*** (-19.62)	−10.3775*** (-15.95)	−11.5463*** (-17.54)	−11.3802*** (-18.29)	−10.3849*** (-15.67)
**Ind**	Yes	Yes	Yes	Yes	Yes	Yes
**Year**	Yes	Yes	Yes	Yes	Yes	Yes
**N**	11398	11398	11398	11398	11398	11398
**adj. *R*^2^**	0.351	0.319	0.356	0.354	0.305	0.346

According to the Model 1-Model 6 in Table 8, the coefficients of M&A goodwill (*GW*) are significantly negative at the level of 1%, which indicate that whether patent application or authorization, invention patent or non-invention patent, M&A goodwill has a significant inhibitory effect on the technological innovation outputs. For patent application, in Model 2, the coefficient of M&A goodwill (*GW*) is -0.9300 and significant at 1% level, while in Model 3, the coefficient is -0.7806 and significant at 1% level, this suggests that goodwill has a greater inhibiting effect on patent applications for inventions. In Model 5, the coefficient of M&A goodwill (*GW*) is -0.9576 and significant at 1% level, while in Model 6 it is -0.8408 and significant at 1% level, this shows that the goodwill of M&A has a greater inhibitory effect on the licensing of invention patents. To sum up, the M&A goodwill significantly inhibits the innovation outputs of corporation, and the inhibition of invention patents is higher than that of non-invention patents.

## 6. Conclusions and implications

Based on panel data of 2,634 Chinese non-financial listed companies from 2007 to 2019, this study examines the impact of M&A goodwill on corporate technological innovation. We find that M&A goodwill has a significant negative correlation with technological innovation, which can be attributed to financial constraints. The ownership difference will result in a negative correlation between M&A goodwill and corporate technological innovation. In addition, stock pledges by controlling shareholders can effectively alleviate the negative impact of M&A goodwill on corporate technological innovation.

### 6.1 Theoretical contributions

Our findings thereby enrich the literature on technological innovation and stock pledges in the following two ways: Firstly, by providing a microcosmic explanation of the negative effects of technological innovation, our research contributes to the literature on the antecedents of technological innovation. Previous literature has emphasized the importance of internal resources in achieving technological innovation [53]. Our findings show that the goodwill generated by M&A consumes internal resources to some extent, thereby limiting the sources of capital available to corporations to invest in technological innovation. Some scholars might argue that the nature of M&A makes it a major strategic decision to survive and thrive [54]. Since goodwill has led to a massive drain of internal resources, it can make up for the lack of innovative resources through external financing. However, the financing abilities of different corporations are different. Compared with non-private corporations, private corporations lack natural advantages in obtaining loans from banks and government subsidies. Through the comparison between private corporations and non-private corporations, we can clearly confirm that although M&A activities consume a large number of internal resources, the resources that their supporters can bring to them make all the difference. As an old Chinese saying goes, “internal worries and external difficulties” make it difficult for private corporations to increase their investment in innovative activities. Therefore, the conclusion of this study strengthens the resource-based theory of corporate technological innovation with a unique research perspective.

Secondly, from the perspective of corporate technological innovation, this study finds that stock pledges by controlling shareholders play a role as a “supporting hand” when M&A goodwill consumes massive resources, which enriches the literature on the economic consequences of stock pledges. Scholars generally hold a negative attitude towards stock pledges by controlling shareholders. They argue that it not only exacerbates conflicts between shareholders and creditors, but also triggers a decline in supplier credit lines [55] and corporate social responsibility performance [56]. Stock pledges by controlling shareholders are essential tools for their own benefit, as many shareholders use shares as collateral for personal loans [57]. Our findings show that the stock pledges by controlling shareholders are not always “bad news”. They can effectively alleviate the financial constraints, and help corporations tide over the crisis. Due to the lack of better financing channels, the controlling shareholders will extend a helping hand to the corporation through stock pledging when the corporation faces financial difficulties. In other words, a special period of stock pledges may be good news for the market, alleviating corporate financial constraints and increasing market value [52]. Hence, the findings of this study are a useful supplement to the existing research on the economic consequences of stock pledges.

### 6.2 Managerial implications

Our findings also have some practical implications. Firstly, our findings show that high-premium M&A activities will have a negative impact on technological innovation, which will not be conducive to the formation of core competitiveness. Consequently, the supervision department should control and supervise the goodwill from the source. On the one hand, the government should supervise the formation of goodwill and information disclosure strictly to prevent and eliminate the irrational behavior of corporations in M&A. On the other hand, the government should perfect the revision of accounting standards for M&A goodwill. Up to now, the measurement of goodwill has always used the “backward crowding” method, that is, to evaluate the fair value of the net assets of the purchased party. This directly results in a high level of impurities and noise in the calculated amount of goodwill, resulting in significant deviations from the economic nature of the goodwill [58]. In the process of revising goodwill accounting standards, regulators should avoid making goodwill a tool of management’s self-interest. Namely, accounting for goodwill should achieve fair measurement while ensuring value relevance and appropriately limiting management’s discretion [59].

Secondly, our research results show that financial constraints will indeed become a restrictive factor for corporate technological innovation inputs. The difficulty and high cost of financing for private corporations in China [30, 32] is a major constraint to the development of technological innovation in private corporations. Therefore, the government should widen the financing channels of private corporations, optimize the financing environment, eliminate the “financial discrimination” of ownership system and alleviate the financing difficulties of private corporations, so as to stimulate the innovation vitality of private corporations, and then successfully achieve the national innovation-driven strategy.

Finally, the government should treat the stock pledges by controlling shareholders as rational behaviors and further improve the information disclosure system for the listed corporations’ pledges. According to the findings of this study, under the special circumstances of M&A, stock pledges by controlling shareholders can effectively alleviate the negative correlation between M&A goodwill and corporate technological innovation, which is of positive significance. But at present, the information disclosure of stock pledges in China is relatively simple, and there is no detailed regulation on the scale and use of the pledged funds, which increases the information asymmetry between the investors and the listed corporations. Therefore, the government should further improve the information disclosure system, correct investors’ bias, and give full play to the financing function of the stock pledges in listed corporations.

### 6.3 Limitations and future research

The limitations of this study are as follows: Firstly, we have confirmed that the heterogeneity of ownership is the primary explanation for the diverse impacts on M&A goodwill and corporate technological innovation. However, the samples are specific to China. Because of the heterogeneity in ownership, the diverse impacts in financing between China’s private and non-private corporations may not occur in other countries, implying that our findings may not be applicable to other countries. From this point, future research could test our conceptual framework in different developing and developed countries. Secondly, for lack of data, we only consider whether the controlling shareholders pledged their stocks, ignoring other characteristics of the stock pledge [55], which are obviously connected with the effect of the stock pledge. Consequently, future researchers could extend the proportion of controlling shareholders’ stock pledges, the timing of the pledge, the risk of transfer control and the pledge terms, which could more profoundly reveal the controlling shareholders’ stock pledges in alleviating the impact of financial constraints on corporate technological innovation. Thirdly, this study only puts innovation inputs and outputs into the theoretical framework model, ignoring the impact of M&A goodwill on technological innovation efficiency [60]. In order to systematically understand the potential economic consequences of M&A goodwill on corporate technological innovation, innovation efficiency should also be considered as an important factor in future research.

## Supporting information

S1 Appendix Change of the measurement methods of technological innovation inputs. (DOCX)

S2 Appendix Long-term impact of M&A goodwill on technological innovation inputs. (DOCX)

S3 Appendix Robustness test for other indicator to measure financial constraints. (DOCX)

S4 Appendix Robustness test for replacing missing values of corporate technological innovation with zero. (DOCX)

S5 Appendix Robustness test for hypothesis H4 with Bootstrap method. (DOCX)
