# Supplementary material for: M&A goodwill and corporate technological innovation: The mediating moderating effect of stock pledges
Source: PLoS One. 2022 Aug 29;17(8):e0271214. doi: 10.1371/journal.pone.0271214 (PMC9423683; doi:10.1371/journal.pone.0271214)
Supplement: S1 Appendix — (DOCX) [file pone.0271214.s001.docx]

**S1 Appendix.** **Change of the measurement methods of technological innovation inputs.**

| Variables | All Sample | Private=1 | Private=0 |
| --- | --- | --- | --- |
|  | RD_t_ | RD_t_ | RD_t_ |
| GW | -0.3236^**^ | -0.5761^***^ | 2.2324^***^ |
|  | (-2.01) | (-3.61) | (3.51) |
| Size | 0.9271^***^ | 0.9798^***^ | 0.8786^***^ |
|  | (33.75) | (27.47) | (21.08) |
| Lev | -0.7735^***^ | -0.6382^***^ | -1.0469^***^ |
|  | (-6.19) | (-4.71) | (-4.13) |
| Roa | 1.1698^***^ | 0.8674^***^ | 1.7077^**^ |
|  | (5.00) | (4.08) | (2.57) |
| Age | -0.2207^***^ | -0.2593^***^ | -0.1994^***^ |
|  | (-7.17) | (-6.43) | (-3.23) |
| Board | 0.0021 | 0.1843^*^ | -0.1263 |
|  | (0.02) | (1.65) | (-0.55) |
| Bm | -0.9070^***^ | -1.1772^***^ | -0.5519^***^ |
|  | (-8.49) | (-9.93) | (-3.39) |
| Top1 | -0.2446 | -0.2550 | 0.0070 |
|  | (-1.60) | (-1.55) | (0.02) |
| Grow | -0.0047 | -0.0084 | 0.0007 |
|  | (-0.41) | (-0.66) | (0.04) |
| _cons | -3.5654^***^ | -5.1637^***^ | -2.3778^**^ |
|  | (-5.90) | (-6.08) | (-2.56) |
| Ind | Yes | Yes | Yes |
| Year | Yes | Yes | Yes |
| *N* | 14186 | 9053 | 5133 |
| adj. *R*^2^ | 0.504 | 0.489 | 0.550 |

Notes：T-statistics in parentheses are one the basis of standard errors clustered by firms and robust to heteroscedasticity. *, ** and *** respectively denote the significance on the basis of two-tailed t-tests at or below 10%, 5%, and 1% level.
