# Supplementary material for: M&A goodwill and corporate technological innovation: The mediating moderating effect of stock pledges
Source: PLoS One. 2022 Aug 29;17(8):e0271214. doi: 10.1371/journal.pone.0271214 (PMC9423683; doi:10.1371/journal.pone.0271214)
Supplement: S2 Appendix — (DOCX) [file pone.0271214.s002.docx]

**S2 Appendix.** **Long-term impact of M＆A goodwill on technological innovation inputs.**

| Variables | All Sample | | Private=1 | | Private=0 | |
| --- | --- | --- | --- | --- | --- | --- |
|  | Model 1 | Model 2 | Model 3 | Model 4 | Model 5 | Model 6 |
|  | RD_t+1_ | RD_t+2_ | RD_t+1_ | RD_t+2_ | RD_t+1_ | RD_t+2_ |
| GW | -0.0161^***^ | -0.0122^**^ | -0.0200^***^ | -0.0156^***^ | 0.0276 | 0.0347 |
|  | (-3.81) | (-2.46) | (-4.45) | (-3.01) | (1.60) | (1.63) |
| Size | 0.0003 | 0.0002 | 0.0017^**^ | 0.0012 | -0.0015^***^ | -0.0013^***^ |
|  | (0.60) | (0.38) | (2.22) | (1.34) | (-3.08) | (-2.63) |
| Lev | -0.0048^**^ | -0.0045^**^ | -0.0071^***^ | -0.0065^**^ | -0.0030 | -0.0030 |
|  | (-2.56) | (-2.18) | (-2.92) | (-2.34) | (-1.03) | (-0.94) |
| Roa | 0.0301^***^ | 0.0381^***^ | 0.0249^***^ | 0.0377^***^ | 0.0358^***^ | 0.0349^***^ |
|  | (5.45) | (5.20) | (4.04) | (3.90) | (3.87) | (3.51) |
| Age | -0.0018^***^ | -0.0011^**^ | -0.0024^***^ | -0.0011 | -0.0033^***^ | -0.0037^***^ |
|  | (-3.31) | (-1.99) | (-2.81) | (-1.11) | (-3.20) | (-3.23) |
| Board | 0.0014 | 0.0013 | 0.0034 | 0.0039 | -0.0018 | -0.0037 |
|  | (0.61) | (0.52) | (1.06) | (1.04) | (-0.53) | (-0.94) |
| Bm | -0.0174^***^ | -0.0169^***^ | -0.0253^***^ | -0.0210^***^ | -0.0090^***^ | -0.0118^***^ |
|  | (-6.47) | (-7.27) | (-8.37) | (-6.40) | (-2.79) | (-3.60) |
| Top1 | -0.0036 | -0.0036 | -0.0053^*^ | -0.0046 | -0.0002 | -0.0008 |
|  | (-1.42) | (-1.30) | (-1.69) | (-1.37) | (-0.05) | (-0.18) |
| Grow | -0.0000 | -0.0000 | 0.0001 | 0.0001 | -0.0001 | -0.0000 |
|  | (-0.24) | (-0.13) | (0.21) | (0.21) | (-0.66) | (-0.24) |
| _cons | 0.0085 | 0.0096 | -0.0181 | -0.0148 | 0.0455^***^ | 0.0493^***^ |
|  | (0.98) | (1.07) | (-1.18) | (-0.91) | (4.13) | (4.10) |
| Ind | Yes | Yes | Yes | Yes | Yes | Yes |
| Year | Yes | Yes | Yes | Yes | Yes | Yes |
| *N* | 10689 | 8470 | 6855 | 5361 | 3834 | 3109 |
| adj. *R*^2^ | 0.282 | 0.275 | 0.262 | 0.251 | 0.320 | 0.323 |

Notes：T-statistics in parentheses are one the basis of standard errors clustered by firms and robust to heteroscedasticity. *, ** and *** respectively denote the significance on the basis of two-tailed t-tests at or below 10%, 5%, and 1% level.
