# Supplementary material for: M&A goodwill and corporate technological innovation: The mediating moderating effect of stock pledges
Source: PLoS One. 2022 Aug 29;17(8):e0271214. doi: 10.1371/journal.pone.0271214 (PMC9423683; doi:10.1371/journal.pone.0271214)
Supplement: S3 Appendix — (DOCX) [file pone.0271214.s003.docx]

**S3 Appendix.** **Robustness test for other indicator to measure financial constraints.**

| Variables | All sample | | | Private=1 | | | Private=0 | | |
| --- | --- | --- | --- | --- | --- | --- | --- | --- | --- |
|  | Model 1 | Model 2 | Model 3 | Model 4 | Model 5 | Model 6 | Model 7 | Model 8 | Model 9 |
|  | RD | KZ | RD | RD | KZ | RD | RD | KZ | RD |
| GW | -0.0244^***^ | 0.6573^***^ | -0.0240^***^ | -0.0284^***^ | 0.5949^***^ | -0.0281^***^ | 0.0153 | 1.3421^**^ | 0.0163 |
|  | (-6.73) | (3.21) | (-6.65) | (-7.25) | (2.67) | (-7.20) | (1.26) | (2.49) | (1.35) |
| KZ |  |  | -0.0006^***^ |  |  | -0.0006^***^ |  |  | -0.0007^**^ |
|  |  |  | (-3.20) |  |  | (-2.62) |  |  | (-2.10) |
| Size | 0.0004 | -0.2536^***^ | 0.0003 | 0.0014^**^ | -0.3463^***^ | 0.0012^*^ | -0.0009^**^ | -0.2064^***^ | -0.0011^**^ |
|  | (1.11) | (-9.57) | (0.72) | (2.01) | (-9.15) | (1.70) | (-2.15) | (-6.05) | (-2.45) |
| Lev | -0.0048^***^ | 6.2499^***^ | -0.0012 | -0.0064^***^ | 6.6218^***^ | -0.0027 | -0.0038 | 5.5274^***^ | 0.0003 |
|  | (-2.87) | (50.99) | (-0.59) | (-2.96) | (41.28) | (-1.04) | (-1.45) | (31.33) | (0.10) |
| Roa | 0.0236^***^ | -8.3913^***^ | 0.0187^***^ | 0.0200^***^ | -7.5240^***^ | 0.0157^***^ | 0.0310^***^ | -10.7355^***^ | 0.0230^***^ |
|  | (5.22) | (-17.83) | (4.04) | (3.83) | (-13.78) | (3.00) | (3.83) | (-15.01) | (2.83) |
| Age | -0.0023^***^ | 0.2786^***^ | -0.0022^***^ | -0.0027^***^ | 0.3106^***^ | -0.0025^***^ | -0.0035^***^ | 0.1616^***^ | -0.0034^***^ |
|  | (-4.50) | (9.31) | (-4.13) | (-3.25) | (6.93) | (-2.99) | (-3.62) | (3.48) | (-3.52) |
| Board | 0.0012 | -0.1598^*^ | 0.0011 | 0.0030 | -0.1802 | 0.0029 | -0.0012 | -0.2578^**^ | -0.0014 |
|  | (0.59) | (-1.79) | (0.54) | (1.09) | (-1.52) | (1.06) | (-0.39) | (-1.97) | (-0.46) |
| Bm | -0.0204^***^ | -0.7491^***^ | -0.0209^***^ | -0.0280^***^ | -0.7899^***^ | -0.0285^***^ | -0.0110^***^ | -0.5259^***^ | -0.0114^***^ |
|  | (-8.81) | (-5.70) | (-8.82) | (-10.30) | (-5.88) | (-10.44) | (-3.98) | (-2.60) | (-4.04) |
| Top1 | -0.0034 | -0.5738^***^ | -0.0037 | -0.0049^*^ | -0.8819^***^ | -0.0054^*^ | -0.0007 | -0.4015^**^ | -0.0010 |
|  | (-1.48) | (-4.49) | (-1.63) | (-1.71) | (-4.64) | (-1.89) | (-0.19) | (-2.29) | (-0.28) |
| Grow | -0.0000 | -0.2721^***^ | -0.0002 | -0.0000 | -0.2310^***^ | -0.0001 | -0.0000 | -0.3608^***^ | -0.0003 |
|  | (-0.32) | (-6.87) | (-1.34) | (-0.10) | (-5.12) | (-0.72) | (-0.18) | (-5.84) | (-1.38) |
| _cons | 0.0056 | 4.2892^***^ | 0.0081 | -0.0134 | 6.2656^***^ | -0.0099 | 0.0349^***^ | 3.8871^***^ | 0.0378^***^ |
|  | (0.72) | (8.42) | (1.05) | (-0.99) | (8.24) | (-0.72) | (3.59) | (5.93) | (3.89) |
| Ind | Yes | Yes | Yes | Yes | Yes | Yes | Yes | Yes | Yes |
| Year | Yes | Yes | Yes | Yes | Yes | Yes | Yes | Yes | Yes |
| *N* | 13989 | 13989 | 13989 | 8882 | 8882 | 8882 | 5107 | 5107 | 5107 |
| adj. *R*^2^ | 0.289 | 0.584 | 0.290 | 0.272 | 0.550 | 0.273 | 0.305 | 0.634 | 0.307 |

Notes：T-statistics in parentheses are one the basis of standard errors clustered by firms and robust to heteroscedasticity. *, ** and *** respectively denote the significance on the basis of two-tailed t-tests at or below 10%, 5%, and 1% level.
