# Supplementary material for: M&A goodwill and corporate technological innovation: The mediating moderating effect of stock pledges
Source: PLoS One. 2022 Aug 29;17(8):e0271214. doi: 10.1371/journal.pone.0271214 (PMC9423683; doi:10.1371/journal.pone.0271214)
Supplement: S4 Appendix — (DOCX) [file pone.0271214.s004.docx]

**S4 Appendix.** **Robustness test for replacing missing values of corporate technological innovation with zero.**

| Variables | All sample | | | Private=1 | | | Private=0 | | |
| --- | --- | --- | --- | --- | --- | --- | --- | --- | --- |
|  | Model 1 | Model 2 | Model 3 | Model 4 | Model 5 | Model 6 | Model 7 | Model 8 | Model 9 |
|  | RD | FC | RD | RD | FC | RD | RD | FC | RD |
| GW | -0.0164^***^ | 0.0913^**^ | -0.0163^***^ | -0.0237^***^ | 0.0759^*^ | -0.0235^***^ | 0.0234^**^ | 0.3797^***^ | 0.0235^**^ |
|  | (-4.96) | (2.40) | (-4.95) | (-6.45) | (1.95) | (-6.44) | (2.30) | (2.98) | (2.31) |
| FC |  |  | -0.0013 |  |  | -0.0035^**^ |  |  | -0.0002 |
|  |  |  | (-1.19) |  |  | (-1.99) |  |  | (-0.19) |
| Size | 0.0012^***^ | -0.0599^***^ | 0.0011^***^ | 0.0026^***^ | -0.0077 | 0.0026^***^ | 0.0000 | -0.0877^***^ | -0.0000 |
|  | (4.19) | (-7.85) | (3.71) | (5.04) | (-1.47) | (5.00) | (0.03) | (-8.72) | (-0.04) |
| Lev | -0.0069^***^ | 0.0309 | -0.0069^***^ | -0.0104^***^ | -0.0054 | -0.0104^***^ | -0.0035^**^ | 0.1121^***^ | -0.0035^**^ |
|  | (-5.33) | (1.31) | (-5.24) | (-6.14) | (-0.26) | (-6.14) | (-2.26) | (3.27) | (-2.23) |
| Roa | 0.0060 | 0.1376 | 0.0062 | 0.0040 | 0.0303 | 0.0041 | 0.0128^***^ | 0.5553^***^ | 0.0130^***^ |
|  | (1.28) | (1.28) | (1.28) | (0.98) | (0.70) | (0.97) | (2.82) | (4.84) | (2.72) |
| Age | -0.0042^***^ | 0.1466^***^ | -0.0040^***^ | -0.0047^***^ | 0.1140^***^ | -0.0043^***^ | -0.0037^***^ | 0.1954^***^ | -0.0037^***^ |
|  | (-11.53) | (26.51) | (-9.63) | (-8.27) | (20.25) | (-6.82) | (-5.93) | (16.89) | (-5.82) |
| Board | 0.0007 | 0.0316^*^ | 0.0008 | 0.0027 | 0.0358^*^ | 0.0028 | -0.0003 | 0.0554^**^ | -0.0003 |
|  | (0.54) | (1.78) | (0.56) | (1.20) | (1.84) | (1.24) | (-0.19) | (1.98) | (-0.18) |
| Bm | -0.0175^***^ | 0.1663^***^ | -0.0173^***^ | -0.0255^***^ | 0.1222^***^ | -0.0250^***^ | -0.0100^***^ | 0.1894^***^ | -0.0099^***^ |
|  | (-10.11) | (8.10) | (-10.01) | (-10.67) | (6.06) | (-10.61) | (-5.21) | (6.42) | (-5.23) |
| Top1 | -0.0025 | -0.1166^***^ | -0.0026 | -0.0030 | 0.0279 | -0.0029 | -0.0009 | -0.1987^***^ | -0.0010 |
|  | (-1.56) | (-4.31) | (-1.64) | (-1.32) | (0.95) | (-1.27) | (-0.47) | (-4.76) | (-0.47) |
| Grow | 0.0000 | 0.0016 | 0.0000 | 0.0000 | 0.0010 | 0.0000 | 0.0000 | -0.0008 | 0.0000 |
|  | (0.28) | (1.22) | (0.31) | (0.10) | (0.81) | (0.14) | (0.59) | (-0.31) | (0.58) |
| _cons | -0.0119^**^ | 4.3592^***^ | -0.0063 | -0.0401^***^ | 3.3533^***^ | -0.0283^**^ | 0.0098 | 4.7334^***^ | 0.0109 |
|  | (-2.05) | (30.38) | (-0.85) | (-3.81) | (30.34) | (-2.52) | (1.64) | (24.32) | (1.26) |
| Ind | Yes | Yes | Yes | Yes | Yes | Yes | Yes | Yes | Yes |
| Year | Yes | Yes | Yes | Yes | Yes | Yes | Yes | Yes | Yes |
| *N* | 21080 | 21080 | 21080 | 11373 | 11373 | 11373 | 9707 | 9707 | 9707 |
| adj. *R*^2^ | 0.369 | 0.374 | 0.369 | 0.339 | 0.315 | 0.339 | 0.341 | 0.506 | 0.341 |

Notes：T-statistics in parentheses are one the basis of standard errors clustered by firms and robust to heteroscedasticity. *, ** and *** respectively denote the significance on the basis of two-tailed t-tests at or below 10%, 5%, and 1% level.
