# Supplementary material for: M&A goodwill and corporate technological innovation: The mediating moderating effect of stock pledges
Source: PLoS One. 2022 Aug 29;17(8):e0271214. doi: 10.1371/journal.pone.0271214 (PMC9423683; doi:10.1371/journal.pone.0271214)
Supplement: S5 Appendix — (DOCX) [file pone.0271214.s005.docx]

**S5 Appendix.** **Robustness test for hypothesis H4 with Bootstrap method.**

| Model | Indirect Effect | BootSE | BootLLCI | BootULCI |
| --- | --- | --- | --- | --- |
| GW→FC→RD | 0.0005 | 0.0002 | 0.0002 | 0.0009 |
